# SOX18 and SOX30 in NSCLC: The Epigenetic Landscape of Methylation, miRNA Regulation, and Network Crosstalk in Tumor Progression

**DOI:** 10.3390/ijms262311669

**Published:** 2025-12-02

**Authors:** Mateusz Olbromski, Aleksandra Piotrowska, Monika Mrozowska, Alicja Kmiecik, Natalia Glatzel-Plucinska, Agnieszka Gomulkiewicz, Aleksandra Stepien, Klaudia Krawczynska, Piotr Blasiak, Marzenna Podhorska-Okolow, Piotr Dziegiel

**Affiliations:** 1 Division of Histology and Embryology, Department of Human Morphology and Embryology, Faculty of Medicine, Wroclaw Medical University, 50-368 Wroclaw, Poland; aleksandra.piotrowska@umw.edu.pl (A.P.); monika.mrozowska@umw.edu.pl (M.M.); alicja.kmiecik@umw.edu.pl (A.K.); natalia.glatzel-plucinska@umw.edu.pl (N.G.-P.); agnieszka.gomulkiewicz@umw.edu.pl (A.G.); aleksandra.stepien@umw.edu.pl (A.S.); klaudia.krawczynska@umw.edu.pl (K.K.); piotr.dziegiel@umw.edu.pl (P.D.); 2 Department and Clinic of Thoracic Surgery, Wroclaw Medical University, Grabiszynska 105, 53-439 Wroclaw, Poland; piotr.blasiak@umw.edu.pl; 3 Lower Silesian Center of Oncology, Pulmonology and Hematology, Hirszfelda 12, 53-413 Wroclaw, Poland; 4 Division of Ultrastructural Research, Wroclaw Medical University, 50-368 Wroclaw, Poland; marzenna.podhorska-okolow@umw.edu.pl; 5 Department of Human Biology, Faculty of Physiotherapy, Wroclaw University of Health and Sport Sciences, 51-612 Wroclaw, Poland

**Keywords:** lung cancer, angiogenesis, non-small cell lung cancer (NSCLC), lung squamous cell carcinoma (LSCC), lung adenocarcinoma, LUAD, SOX, SOX7, SOX17, SOX18, SOX30, MEF2C, VCAM1, STAT3

## Abstract

SOX (SRY-related HMG-box) transcription factors are key regulators of embryogenesis and vascular development, with emerging roles in cancer biology. In non-small-cell lung cancer (NSCLC), the contributions of SOX18 and SOX30 remain insufficiently understood, particularly regarding their epigenetic regulation and network interactions with angiogenic and immune-modulatory pathways. We examined 800 NSCLC specimens (400 lung adenocarcinomas, 400 squamous cell carcinomas) using immunohistochemistry, RT-qPCR, Western blotting, and spatial transcriptomics to profile SOX18, SOX30, and related signaling partners (SOX7, SOX17, MEF2C—Myocyte Enhancer Factor 2C, VCAM1—Vascular Cell Adhesion Molecule 1, p-STAT3—Signal Transducer and Activator of Transcription 3). Epigenetic regulation was assessed via droplet digital methylation-specific PCR of promoter CpG islands, while functional validation employed adenoviral delivery of hsa-miR-24-3p in NSCLC cell lines and 3D spheroid cultures. SOX18 protein was markedly overexpressed in both NSCLC subtypes, despite reduced transcript levels and consistent promoter hypermethylation, suggesting post-transcriptional regulation. In contrast, SOX30 expression was uniformly downregulated at both mRNA and protein levels, frequently linked to promoter hypermethylation, especially in squamous carcinoma. Spatial transcriptomics revealed SOX18 enrichment at tumor cores and invasive borders, co-localizing with MEF2C, VCAM1, and p-STAT3 in vascular and stromal niches, while SOX30 expression remained low across all tumor regions. Functional assays demonstrated that hsa-miR-24-3p suppressed SOX18 expression and partially modulated SOX30 and MEF2C, reinforcing a miRNA-driven regulatory axis. In summary, SOX18 and SOX30 play divergent roles in NSCLC progression: SOX18 functions as a pro-oncogenic factor driving angiogenesis and tumor–stroma interactions, while SOX30 acts as an epigenetically silenced tumor suppressor. Regulation of SOX18 by miR-24-3p highlights a potential therapeutic vulnerability. These findings underscore the significance of SOX transcription factors as biomarkers and potential targets for novel treatment strategies in NSCLC.

## 1. Introduction

Lung cancer remains the leading cause of cancer mortality worldwide. In 2022, it accounted for approximately 2.48 million new cases, representing 12.4% of all cancer diagnoses, and caused 1.82 million deaths, or 18.7% of global cancer mortality [1,2,3]. Among emerging molecular targets in NSCLC, the SOX (SRY-related HMG-box) family of transcription factors has garnered increasing attention. In recent years, their role in tumors has been intensively studied, resulting in the demonstration of these transcription factors’ participation in the pathogenesis of many malignant tumors [4].

The SOX family comprises over 20 members grouped from A to H based on sequence homology and functional domains [5,6]. Group F proteins (SOX7, SOX17, SOX18) are primarily involved in the development of the cardiovascular system during embryogenesis [7]. SOX7 [8], SOX17 [9], and SOX18 [10,11,12] are involved in the same pathways as the vascular endothelial growth factors (VEGFs), indicating their participation in the development of the cardiovascular system and lymphangiogenesis [7,13,14,15].

In this context, SOX18 has emerged as a key regulatory node in tumor angiogenesis and immune modulation. It physically interacts with MEF2C, a transcription factor involved in cardiovascular and endothelial development, forming cooperative complexes that regulate genes essential for vascular remodeling [10,16]. In addition to MEF2C, SOX18 regulates the expression of VCAM1 (Vascular Cell Adhesion Molecule 1), a gene critical for leukocyte adhesion and transmigration. SOX18 upregulates VCAM1 transcription under inflammatory and angiogenic stimuli, suggesting its role in modulating the tumor microenvironment through endothelial activation and immune cell recruitment [17,18]. This could be crucial not only in metastasis processes of cancer cells but also in tumor angiogenesis. Moreover, emerging data suggest a context-dependent crosstalk between SOX18 and the STAT3 signaling pathway, a key regulator of cancer cell survival, proliferation, and immune evasion. Although direct binding between SOX18 and STAT3 has not been fully elucidated, SOX18 has been shown to modulate gene expression patterns overlapping with STAT3 targets, suggesting a convergent transcriptional axis.

The SOX30 protein (SRY-box containing gene 30), the only member of the group H, has been characterized in a few species [19,20]. Experimental studies demonstrated that SOX30 can promote apoptosis by directly activating *TP53* transcription, and its higher expression correlates with improved patient survival [21,22,23,24,25,26].

Experimental studies have shown that restoration of SOX30 enhances apoptotic signaling and reduces cellular proliferation, while loss or inhibition of SOX30 diminishes p53 activity and favors tumorigenic behavior [23,24,25,26]. Although SOX30 contributes to tumor suppression, current evidence does not support its role as a singular ‘key’ regulator of tumorigenesis. Moreover, data on a direct involvement of SOX30 in angiogenesis are limited; if present, such effects are likely indirect, potentially mediated through p53-dependent pathways and interactions with inflammatory networks such as p-STAT3–VCAM1, consistent with the correlations observed in this study.

The role of SOX proteins, including SOX7, SOX17, SOX18, and SOX30 in lung cancer is not well known. Crucially, both SOX18 and SOX30 are subject to epigenetic regulation. Hypermethylation of promoter CpG islands within these genes has been documented in lung cancer cell lines and primary tumors, correlating with reduced mRNA expression [27,28,29].

In addition to DNA methylation, microRNAs (miRNAs) have been implicated in the post-transcriptional regulation of SOX18 and SOX30 [30,31,32,33,34,35]. In the case of SOX18, the observed discrepancies between mRNA and protein levels in NSCLC suggest regulation by miRNAs—specifically hsa-miR-7a and hsa-miR-24-3p—a mechanism also seen in other types of cancer.

Taken together, the epigenetic silencing and post-transcriptional modulation of SOX18 and SOX30 may play a crucial role in the initiation and progression of LUAD and LSCC. The aim of this study is to investigate both the epigenetic modifications of *SOX18* and *SOX30* through analysis of CpG methylation, as well as the post-transcriptional regulation of *SOX18* by miRNA-24-3p, based on our findings, indicating its inhibitory effect. We focused on miR-24-3p because our previous work demonstrated its strong regulatory impact on SOX factors and its functional relevance in NSCLC biology [30,32]. In contrast, miR-7a, although an important tumor-suppressive miRNA, regulates multiple essential oncogenic pathways, and its artificial down- or up-regulation may induce broad, undesirable pleiotropic effects, as reported in several studies. Therefore, miR-24-3p represented a more specific and mechanistically justified candidate for targeted functional evaluation in this study.

## 2. Results

### 2.1. SOX Proteins in the Landscape of NSCLC Progression

**Figure 1:**
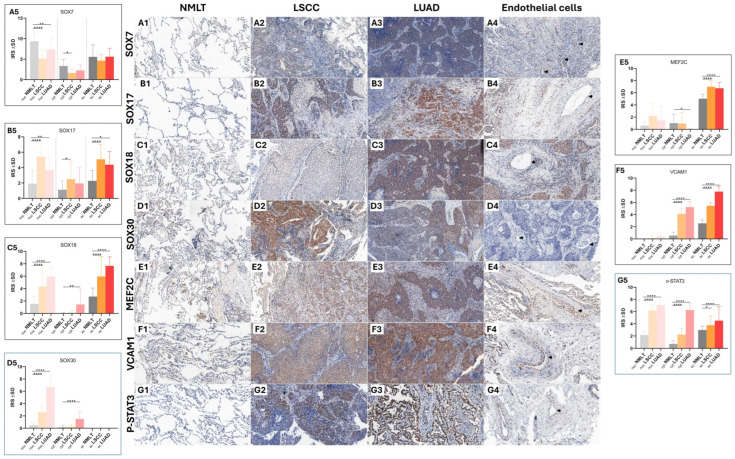
Immunohistochemical analysis of SOX7, SOX17, SOX18, SOX30, MEF2C, VCAM1, and p-STAT3 expression in non-malignant lung tissue (NMLT; *n* = 55) and non-small-cell lung cancer (NSCLC) samples, including squamous cell carcinoma (LSCC; *n* = 400) and adenocarcinoma (LUAD; *n* = 400). All analyzed markers demonstrated nuclear, cytoplasmic, and endothelial localization (**A1**–**G4**). With the exception of SOX30, all markers showed significantly increased expression in the nuclear and endothelial compartments of both LSCC and LUAD samples compared with NMLT (*p* < 0.0001). Subfigures (**A5**–**G5**) present the quantitative immunoreactivity scores (IRS ± SD) for the corresponding markers: (**A5**) SOX7, (**B5**) SOX17, (**C5**) SOX18, (**D5**) SOX30, (**E5**) MEF2C, (**F5**) VCAM1, and (**G5**) phosphorylated STAT3 (p-STAT3) in NMLT, LSCC, and LUAD. Statistical significance is indicated as *p* < 0.05 (*), *p* < 0.01 (**), and *p* < 0.0001 (****). Arrows indicate representative positively stained endothelial cells. Original magnification: ×200.

Immunohistochemical analysis showed that the markers SOX7, SOX17, SOX18, SOX30, MEF2C, VCAM1, and p-STAT3 were present in samples from LUAD, LSCC, and non-malignant lung tissue (NMLT) (Figure 1(A1–G4)).

A detailed summary of how frequently each marker appeared in these cellular locations across the sample groups can be found in Table 1, which outlines the distribution patterns in a clear and accessible format.

Statistical analysis revealed that SOX7 exhibited significantly higher nuclear expression in non-malignant lung tissue (NMLT) compared to both LSCC (*p* < 0.0001) and LUAD (*p* < 0.01) samples, as determined by the Mann–Whitney test (Figure 1(A5)). In contrast, SOX17 showed increased expression in both LSCC and LUAD samples relative to NMLT across all assessed cellular compartments. Specifically, nuclear localization of SOX17 was significantly elevated in LSCC (*p* < 0.0001) and LUAD (*p* < 0.01), while cytoplasmic expression also showed an upward trend (*p* < 0.1).

Additionally, SOX17 expression in vascular endothelial cells was significantly higher in LSCC (*p* < 0.0001) and showed a moderate increase in LUAD (*p* < 0.1) (Figure 1(B5)). SOX18 demonstrated significantly elevated protein expression in the nuclei of both LSCC and LUAD samples compared to non-malignant lung tissue (NMLT) (*p* < 0.0001 for both comparisons; Mann–Whitney test). Additionally, cytoplasmic expression of SOX18 was significantly higher in LUAD cells than in NMLT (*p* < 0.01). Notably, SOX18 expression was also markedly increased in the endothelial cells of tumor-associated blood vessels in both LSCC and LUAD samples when compared to those in NMLT (*p* < 0.0001 for both) (Figure 1(C5)). SOX30 protein expression was significantly elevated in the nuclei of both LSCC and LUAD samples, as well as in the cytoplasm of LUAD cells, compared to non-malignant lung tissue (NMLT) (*p* < 0.0001 for all comparisons; Mann–Whitney test) (Figure 1(D5)). MEF2C expression was significantly higher in the endothelial cells of blood vessels in both LSCC and LUAD samples compared to non-malignant lung tissue (NMLT) (*p* < 0.0001 for both comparisons; Figure 1(D5)). VCAM1 expression was significantly elevated in both the cytoplasm of LSCC and LUAD cells and in the endothelial cells of tumor-associated blood vessels when compared to non-malignant lung tissue (NMLT) (*p* < 0.0001 for all comparisons; Figure 1(E5)). p-STAT3 expression was significantly higher in the nuclei of both LSCC and LUAD samples compared to non-malignant lung tissue (NMLT) (*p* < 0.0001 for both; Mann–Whitney test). Similarly, cytoplasmic expression levels were elevated in LSCC and LUAD samples relative to NMLT (*p* < 0.0001 for both). Increased expression was also observed in the endothelial cells of blood vessels, with statistical significance in LSCC (*p* < 0.1) and LUAD (*p* < 0.0001) cases (Figure 1(F5)). We observed significantly increased levels of p-STAT3 in the cytoplasm, nuclei, and endothelial cells in LUAD (*p* < 0.0001, *p* < 0.0001, and *p* < 0.0001, respectively) and LSCC (*p* < 0.0001, *p* < 0.0001, and *p* < 0.1) compared with NMLT tissue (Figure 1(G5)).

**Figure 2:**
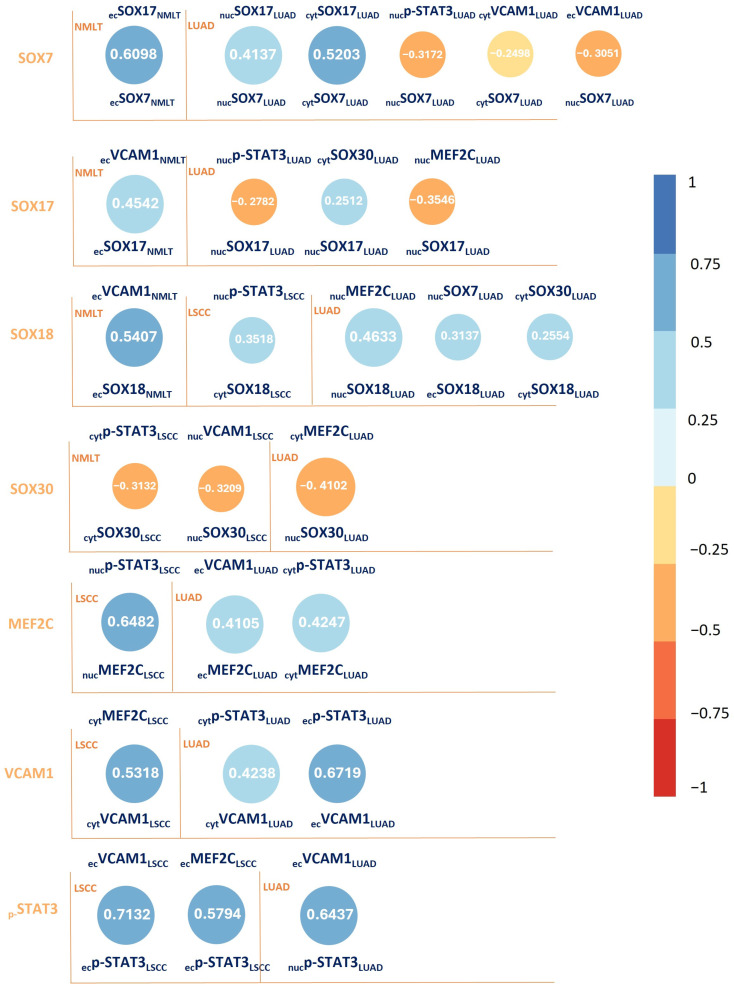
Spearman correlation matrix of the investigated markers: SOX7, SOX17, SOX18, SOX30, MEF2C, VCAM1, and p-STAT3. Bubble plots display correlation coefficients ranging from −1 to 1. Dark blue bubbles represent positive correlations, while dark red bubbles indicate negative correlations. The size of each bubble reflects the strength of the correlation: larger bubbles correspond to stronger correlations (r values closer to ±1), whereas smaller bubbles indicate weaker associations (r values near 0). Numerical values displayed within the bubbles represent the Spearman correlation coefficient (r).

The Spearman correlation analysis revealed both strong positive and negative associations among the studied proteins, as illustrated in Figure 2.

### 2.2. Distinct Patterns of SOX Family Gene Expression in NSCLC Revealed by RT-qPCR and Western Blotting

**Figure 3:**
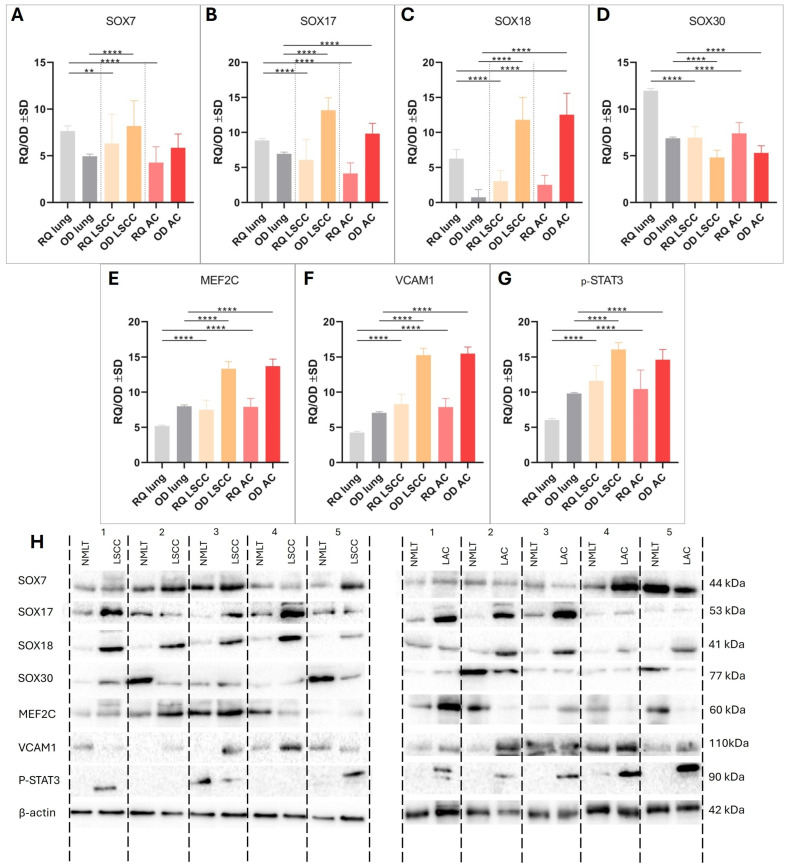
mRNA and protein expression levels of SOX7 (**A**), SOX17 (**B**), SOX18 (**C**), SOX30 (**D**), MEF2C (**E**), VCAM1 (**F**), and STAT3 (**G**) in 50 LSCC and 50 LUAD tumor samples, along with matched non-malignant lung tissue (NMLT) controls. Relative mRNA expression levels were assessed using quantitative real-time PCR. (**H**) Representative results from 10 matched NSCLC–NMLT sample pairs are shown, indicated by sample numbers above each lane. Densitometric analysis of Western blot bands revealed significantly higher protein expression in NSCLC tissues compared to NMLT for all markers except SOX30. β-actin was used as a loading control. Data are presented as mean ± standard deviation from three independent experiments. **, *p* < 0.01; **** *p*  <  0.0001. The results were identical after three repetitions of this experiment.

Real-time PCR and Western blot analyses revealed distinct expression patterns of SOX family genes and related markers in NSCLC subtypes compared to non-malignant lung tissue (NMLT), as shown in Figure 3. *SOX7* mRNA expression was significantly lower in both LSCC and LUAD samples compared to NMLT (*p* < 0.01 and *p* < 0.0001, respectively); however, SOX7 protein levels were significantly elevated in LSCC (*p* < 0.0001), indicating post-transcriptional regulation (Figure 3A). SOX17 showed significantly increased protein expression in both LSCC and LUAD (*p* < 0.0001), despite no reported mRNA differences (Figure 3B). In contrast, *SOX18* demonstrated reduced mRNA expression in LSCC and LUAD relative to NMLT (both *p* < 0.0001), while its protein levels were significantly elevated in both cancer types (*p* < 0.0001) (Figure 3C). SOX30 expression was consistently downregulated at both the mRNA and protein levels in LSCC and LUAD (all *p* < 0.0001) (Figure 3D). Furthermore, MEF2C, VCAM1, and STAT3 were significantly upregulated at both transcript and protein levels in LSCC and LUAD compared to NMLT (*p* < 0.0001 for all comparisons), underscoring their potential role in NSCLC pathogenesis (Figure 3E–H).

### 2.3. Spatially Resolved Transcriptomics Uncovers the Landscape of SOX and SOX-Related Gene Expression in LSCC and LUAD

**Figure 4:**
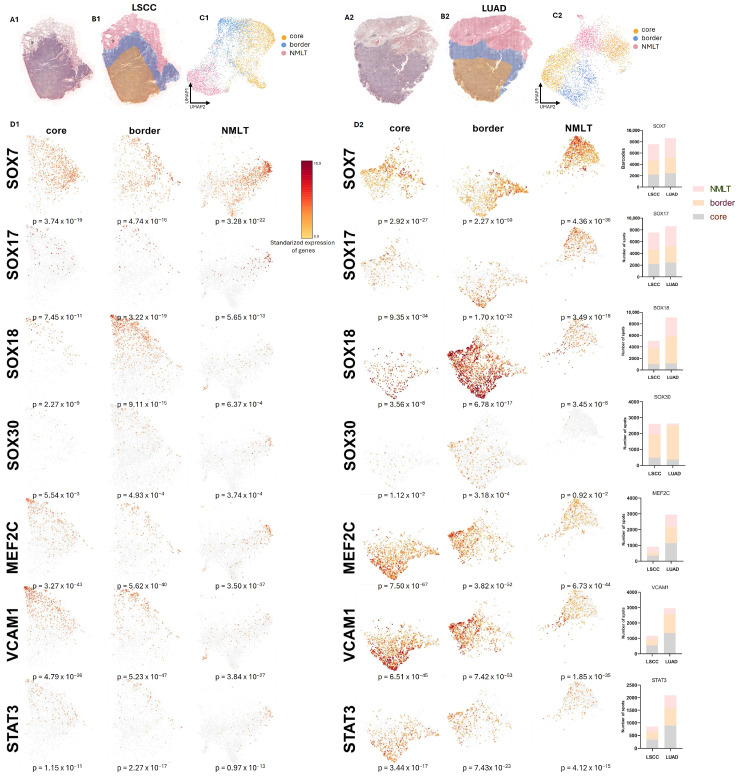
Schematic overview of the spatially resolved transcriptomics (**SRT**) experimental workflow. Representative H&E-stained images of LSCC (**A1**) and LUAD (**A2**) tissue sections used for SRT analysis are shown. Regions corresponding to tumor core, invasive border, and adjacent non-malignant lung tissue (NMLT) were manually annotated and selected for analysis in both LSCC (**B1**) and LUAD (**B2**) samples. UMAP projections of transcriptomic spots, aggregated according to core, border, and NMLT regions, are presented for LSCC (**C1**) and LUAD (**C2**), illustrating distinct spatial transcriptomic clustering patterns. Average standardized expression levels of annotated genes—*SOX7*, *SOX17*, *SOX18*, *SOX30*, *MEF2C*, *VCAM1*, and *STAT3*—in LSCC (**D1**) and LUAD (**D2**) tissue sections. Spatial transcriptomic data reveal coherent expression patterns across distinct anatomical regions, including the tumor core, invasive border, and adjacent non-malignant lung microenvironment. *p*-values indicate the significance of spatial clustering, calculated by comparing the observed distances between spots expressing genes associated with specific GO terms to a null distribution of distances derived from randomly selected spots (Wilcoxon rank-sum test).

We conducted spatial transcriptomics (ST) on two formalin-fixed, paraffin-embedded (FFPE) NSCLC tissue sections—one from a lung squamous cell carcinoma (LSCC) patient and one from a lung adenocarcinoma (LUAD) patient—using the 10x Genomics Visium Spatial Gene Expression platform (Figure 4(A1,A2)).

The resulting dataset captured transcriptomic information from 9478 spatially barcoded spots in the LSCC sample and 9623 spots in the LUAD sample, with expression detected for approximately 18,000 unique genes across both tissues. Sequencing generated high-resolution data from 23,463 spots in the LSCC sample and 22,363 in the LUAD sample, with a mean post-normalization read depth of 41,423 and 40,371 reads per spot, respectively.

Hematoxylin and eosin (H&E)-stained tissue sections were used to annotate histologically distinct regions—tumor core, invasive border, and adjacent non-malignant lung tissue (NMLT)—which guided spatial interpretation (Figure 4(A1,A2)). Panels B1 and B2 highlight the morphologically distinct regions identified within the tissue sections—specifically, the tumor core (marked in yellow), the invasive tumor border (blue), and the adjacent non-malignant lung tissue (NMLT, red). Corresponding UMAP projections in panels C1 and C2 illustrate how transcriptomic profiles cluster spatially, mirroring these histologically annotated regions and confirming their unique molecular identities.

Spatial gene expression profiles shown in panels D1 and D2, a number of striking patterns emerge. *SOX7* expression is most prominent in the non-malignant lung tissue across both LSCC and LUAD samples, with significantly reduced levels observed within the tumor core and border. This downregulation aligns with *SOX7*’s proposed role in maintaining vascular and epithelial homeostasis, and its known suppression in malignant contexts. In contrast, *SOX17* is upregulated in tumor regions—particularly in LUAD—supporting its emerging role as an oncogenic factor in NSCLC, potentially contributing to stem-like phenotypes and tumor identity.

*SOX18* shows the most pronounced spatial enrichment, with expression sharply concentrated in the tumor core and extending into the invasive border in both cancer subtypes. This spatial trend is consistent with *SOX18*’s involvement in angiogenesis and vascular remodeling within the tumor microenvironment. Meanwhile, *SOX30* exhibits a markedly different pattern: its expression is uniformly low across all regions, with the weakest signals found in tumor tissues.

In addition to the *SOX* genes, the analysis of other related markers reveals further insights. *MEF2C* is predominantly expressed in the tumor core of both LSCC and LUAD, underscoring its association with cancer cell proliferation and its potential regulatory interaction with *STAT3*. *VCAM1* displays a particularly strong signal along the tumor border, suggesting active crosstalk between tumor cells and the surrounding stroma or endothelium—a known function of *VCAM1* in promoting tumor-associated inflammation and cell adhesion. *STAT3* follows a similar trend, with enriched expression throughout the tumor regions and strongest levels observed at the core and border. This pattern is consistent with *STAT3*’s well-documented role in driving oncogenic transcriptional programs and facilitating immune evasion.

### 2.4. Divergent Methylation Patterns of SOX18 and SOX30 in NSCLC Reveal Distinct Epigenetic Mechanisms

**Figure 5:**
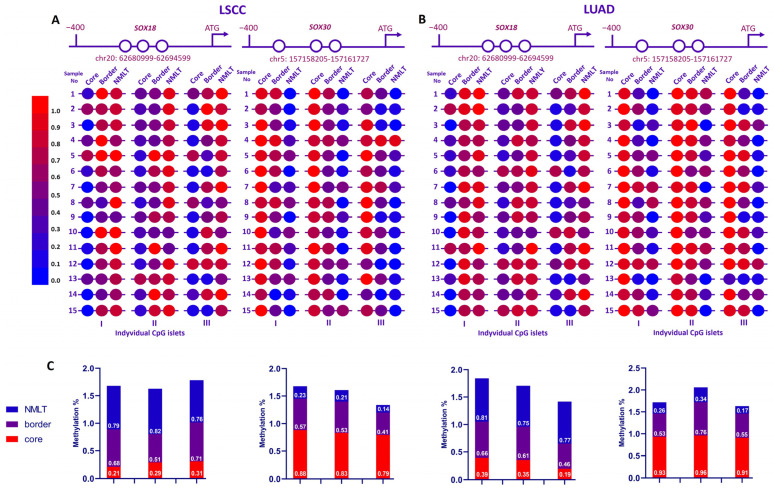
Heatmaps representing methylation levels of individual CpG islets within the *SOX18* and *SOX30* promoter regions in paired tumor core, tumor border, and non-malignant lung tissue (NMLT) from patients with lung squamous cell carcinoma (LSCC) (**A**) and lung adenocarcinoma (LUAD) (**B**). For each gene, three CpG-rich regions (islets I–III) upstream of the ATG start site were assessed using quantitative methylation analysis. (**C**) Methylation levels are color-coded from blue (unmethylated, 0.0) to red (fully methylated, 1.0), as indicated in the scale bar. Each circle represents the mean methylation level of the entire CpG islet (I–III).

As shown in Figure 5A,B, to explore how *SOX18* and *SOX30* are epigenetically regulated in non-small-cell lung cancer (NSCLC), we analyzed the methylation status of three distinct CpG islets within their promoter regions across 30 patient samples (LSCC, n = 15; LUAD, n = 15). Each sample included tumor core, border, and matched non-malignant lung tissue (NMLT) from both lung squamous cell carcinoma (LSCC) and lung adenocarcinoma (LUAD) cases.

Across both NSCLC subtypes, *SOX18* promoter methylation was clearly elevated in surrounding lung tissues compared to tumor cores. This trend was especially evident in CpG islets I and II. The heatmaps reveal stronger red tones in NMLT regions, reflecting higher methylation levels. Quantitative analysis further confirmed this difference: core samples consistently showed increased methylation relative to NMLT and tumor border regions.

In contrast, the methylation pattern for *SOX30* showed more variation. In LSCC samples, *SOX30* promoter regions tended to be highly methylated in tumor cores, with levels tapering off toward NMLT. However, in LUAD samples, the methylation landscape was notably more heterogeneous. While some LUAD cores showed strong hypermethylation, others presented more moderate or variable levels. This indicates that *SOX30* methylation is influenced by tumor subtype, and its regulation may differ between squamous and adenocarcinoma histology.

Overall, while both genes displayed promoter hypermethylation in cancerous tissue, SOX18 methylation was more consistent across both LUAD and LSCC, whereas *SOX30* showed greater variability, particularly in LUAD cases.

As shown in Figure 5C, average methylation percentages illustrate these differences clearly. For *SOX18*, core regions had elevated methylation (ranging from ~0.68 to 0.71), while NMLT remained lower (0.76–0.82). *SOX30* showed a wider range, with LSCC cores reaching high methylation levels (~0.88), in contrast to lower values in matched NMLT (~0.14–0.23). LUAD samples again showed greater variation, reinforcing the observation of subtype-dependent regulation.

### 2.5. Adenoviral Vector Delivery of hsa-miR-24-3p Suppresses SOX18 and Modulates Its Regulatory Network

**Figure 6:**
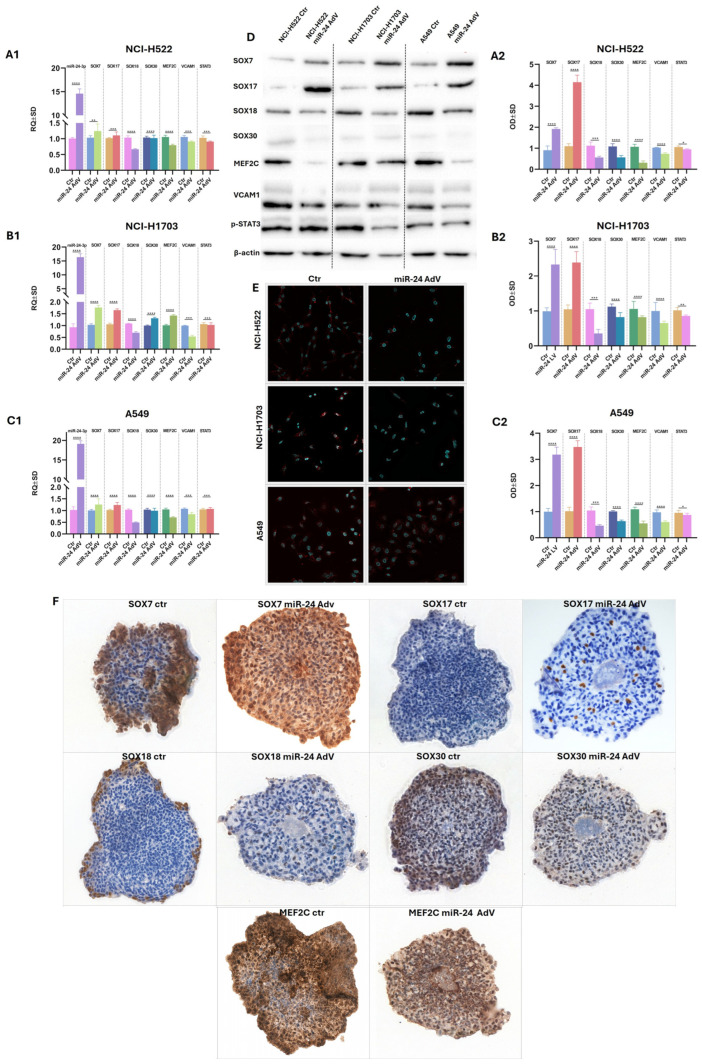
(**A1**–**C1**) Quantitative RT-PCR analysis of gene expression in NCI-H522 (**A1**), NCI-H1703 (**B1**), and A549 (**C1**) cell lines following adenoviral overexpression of hsa-miR-24-3p. The expressions of *SOX7*, *SOX17*, *SOX18*, *SOX30*, *MEF2C*, *VCAM1*, and *STAT3* was assessed, demonstrating significant downregulation of *SOX18* transcripts after miR-24-3p transduction. Data are presented as mean ± SD; statistical significance is indicated. (**A2**–**C2**) Densitometric analysis of immunoblotting corresponding to the transcripts measured in (**A1**–**C1**). Protein levels reflect a pattern consistent with the transcriptomic data, confirming suppression of SOX18 and associated gene products following miR-24-3p overexpression. (**D**) Representative Western blot showing expression levels of SOX7, SOX17, SOX18, SOX30, MEF2C, VCAM1, and STAT3 in control and miR-24-3p-transduced cells. β-actin serves as a loading control. Protein suppression of SOX18 is observed upon miR-24-3p overexpression. (**E**) Confocal immunofluorescence microscopy of SOX18 in NCI-H522, NCI-H1703, and A549 cells, comparing control and miR-24-3p-transduced conditions. Decreased SOX18 nuclear signal is evident in all lines post-transduction. (**F**) Immunohistochemical staining of 3D spheroid cultures of A549 and HUVEC lines, showing SOX7, SOX17, SOX18, SOX30, and MEF2C expression in control and miR-24-3p-overexpressing cells. Notably, peripheral nuclear SOX18 staining is diminished in miR-24-3p-treated spheroids, indicating targeted inhibition at the protein level. *, *p* < 0.1, **, *p* < 0.01, ***, *p* < 0.001, **** *p* < 0.0001.

To elucidate the regulatory influence of miR-24-3p on SOX18 and SOX-related proteins in NSCLC, we employed adenoviral delivery of miR-24-3p into three NSCLC cell lines: NCI-H522, NCI-H1703, and A549—using the AdmRa-hsa-miR-24 viral vector obtained from ABM. Quantitative RT-PCR analysis (Figure 6(A1–C1)) revealed robust and consistent downregulation of SOX18 transcripts across all three cell lines following miR-24-3p delivery (Figure 6(A1–C1)), confirming a strong post-transcriptional regulatory effect. Simultaneously, we observed moderate but reproducible suppression of SOX30 and *MEF2C* mRNAs, while expression of *SOX7*, *SOX17*, *VCAM1*, and *STAT3* exhibited cell line-dependent variability, indicating that miR-24-3p selectively targets specific components of the SOX-related regulatory network—SOX18. Densitometric quantification (Figure 6(A2–C2)) aligned closely with mRNA data, indicating coordinated transcriptional and translational regulation. Notably, SOX30 and MEF2C protein levels also decreased, although the reduction was smaller than that observed for SOX18. Transcriptional trends were substantiated at the protein level by Western blotting (Figure 6D), which confirmed marked depletion of SOX18 across all cell lines post transduction.

Immunofluorescence imaging using confocal microscopy (Figure 6E) provided spatial validation of these findings, revealing strong nuclear SOX18 localization in control cells and a near-complete loss of nuclear signal following miR-24-3p overexpression. To further corroborate these effects in a 3D context, we analyzed tumor spheroids by immunohistochemistry (Figure 6F). The spheroids were composed of A549 cells co-cultured with HUVECs, serving as a vascular scaffold. In untreated spheroids, SOX18 expression was predominantly localized to peripheral regions, showing strong nuclear staining. This peripheral signal was significantly diminished in spheroids exposed to miR-24-3p, mirroring the reductions observed in 2D cultures. Similar expression patterns were noted for SOX7, SOX17, SOX30, and MEF2C, reinforcing the broader regulatory impact of miR-24-3p on SOX-family members.

Due to the limited number of spheroid samples available, we were unable to extend immunohistochemical analysis to additional markers beyond the selected SOX-related proteins. Furthermore, we could not definitively assess the influence of SOX18 or miR-24-3p on the HUVEC scaffold component, as the aggressive overgrowth of A549 cells masked or displaced the endothelial population within the 3D structures.

Collectively, these findings demonstrate that hsa-miR-24-3p acts as a potent negative regulator of SOX18 expression at both the transcript and protein levels and partially reshapes the associated SOX–MEF2C regulatory network in NSCLC cells.

## 3. Discussion

In this study, we comprehensively examined the expression patterns, regulatory mechanisms, and potential functional relevance of SOX family transcription factors—particularly SOX7, SOX17, SOX18, and SOX30—as well as their molecular interactors (MEF2C, VCAM1, and STAT3) in the context of non-small-cell lung cancer (NSCLC). Through a combination of immunohistochemistry, molecular profiling, spatial transcriptomics, epigenetic analysis, and functional assays, we delineated a complex yet coherent landscape of SOX-related signaling across histologically distinct NSCLC subtypes, namely lung squamous cell carcinoma (LSCC) and lung adenocarcinoma (LUAD).

Our immunohistochemical data revealed distinct expression profiles across tumor and non-tumor compartments, pointing to both subtype-specific and shared regulatory pathways. One of the most important findings was the downregulation of nuclear SOX7 in tumor tissue compared to non-malignant lung samples, in alignment with previous reports implicating SOX7 as a tumor suppressor in various cancers, including lung, breast, and colon carcinomas. The observed negative correlation between SOX7 and oncogenic markers such as STAT3 and VCAM1, particularly in LUAD, adds further weight to the hypothesis that SOX7 exerts anti-proliferative or anti-angiogenic functions in the pulmonary epithelium, possibly by antagonizing transcriptional programs that drive inflammation and invasion.

In contrast, SOX17 and SOX18 exhibited pronounced nuclear and endothelial expressions in tumor tissues, with SOX17 particularly enriched in LUAD and SOX18 in both LUAD and LSCC. SOX18, a critical regulator of angiogenesis during embryonic development, appears repurposed in the tumor setting to facilitate neovascularization and stromal remodeling. Its strong spatial enrichment at the tumor core and invasive front, as shown by our spatial transcriptomic maps, provides spatial evidence supporting its function in reshaping the tumor microenvironment—likely through vascular expansion and endothelial–tumor crosstalk.

These oncogenic features of SOX17 and SOX18 are supported by their significant co-expression with endothelial and inflammatory mediators such as VCAM1 and STAT3, both of which are known to drive tumor-associated angiogenesis, immune evasion, and metastatic potential. The positive correlation between SOX18 and MEF2C in LUAD, and the strong endothelial co-expression of SOX18 with VCAM1, suggests a coordinated transcriptional module possibly orchestrating endothelial activation in tumor-associated vasculature. MEF2C, previously implicated in hematopoietic malignancies and increasingly recognized in solid tumors, may act downstream or in parallel with STAT3, forming a signaling axis that contributes to NSCLC progression through modulation of vascular and immune niches.

SOX30, in contrast to SOX17 and SOX18, exhibited suppressed expression at both mRNA and protein levels across tumor regions. This consistent downregulation, coupled with negative correlations to STAT3 and VCAM1 expression, especially in LSCC, supports the notion of SOX30 as a tumor suppressor. Prior studies have shown SOX30’s involvement in promoting differentiation and apoptosis in testicular and lung tissues. Its epigenetic silencing through promoter hypermethylation, which we observed particularly in LSCC, aligns with mechanisms described in other cancers and underscores the gene’s vulnerability to transcriptional repression during tumorigenesis.

Interestingly, our methylation analysis revealed differential regulatory patterns between *SOX18* and *SOX30*. *SOX18* showed consistent promoter hypermethylation across tumor samples, despite its protein overexpression. This apparent paradox may reflect a more complex post-transcriptional regulatory framework, possibly involving alternative transcripts, compensatory feedback loops, or microRNA-mediated modulation. Indeed, our functional studies using adenoviral delivery of hsa-miR-24-3p into NSCLC cell lines demonstrated potent downregulation of SOX18 at both mRNA and protein levels, with additional moderate effects on SOX30 and MEF2C. These results suggest that miR-24-3p may target a specific regulatory module in NSCLC, highlighting its potential as a therapeutic agent or biomarker, particularly in tumors with SOX18-driven angiogenic programs.

Spatial transcriptomics provided critical spatial context to our molecular findings. The enriched expression of *STAT3*, *VCAM1*, and *MEF2C* at the tumor core and invasive front underscores their role in shaping the tumor–stroma interface. The strong co-expression of these genes with *SOX18* in vascular regions suggests the existence of a spatially restricted signaling network potentially involved in sustaining tumor-associated vasculature. *VCAM1*’s localization at the tumor border, in particular, may reflect its role in mediating adhesion between tumor and endothelial cells—a key step in metastatic dissemination.

In conclusion, our study highlights the distinct and sometimes opposing roles of SOX family members in NSCLC progression, with SOX7 and SOX30 acting as putative tumor suppressors, while SOX17 and SOX18 may promote oncogenic and angiogenic programs, particularly through cooperation with p-STAT3, MEF2C, and VCAM1. The spatial and epigenetic complexity of their regulation underscores the importance of integrative, multimodal approaches in uncovering novel targets and pathways in lung cancer. Future studies will aim to validate these findings in larger cohorts and functional models, and to explore the therapeutic potential of modulating SOX-dependent signaling, particularly through epigenetic or miRNA-based interventions.

## 4. Materials and Methods

### 4.1. Patients and Clinical Samples

A total of 800 NSCLC samples were obtained from patients treated at the Lower Silesian Centre of Lung Diseases in Wroclaw between 2016 and 2019. The study cohort included 400 adenocarcinomas (LUADs) and 400 squamous cell carcinomas (LSCCs). For each sample, formalin-fixed paraffin-embedded (FFPE) tissue blocks were created. Clinical, pathological, and survival data were sourced from hospital archives and are presented in Table 2.

### 4.2. Cell Lines

The lung cancer cell lines—NCI-H522 (CRL-5810™, lung adenocarcinoma), NCI-H1703 (CRL-5889™, lung squamous cell carcinoma), and A549 (CCL-185™, lung adenocarcinoma), as well as Human Microvascular Endothelial Cells (HMEC-1, PCS-110-010™) and Human Umbilical Vein Endothelial Cells (HUVEC, PCS-100-010™) were obtained from American Type Culture Collection (ATCC^®^, Old Town Manassas, VA, USA). NCI-H1703 and NCI-H522 cells were cultured in RPMI-1640 (ATCC modification, A104910) medium supplemented with 10% fetal bovine serum (FBS) and 1% streptomycin/penicillin solution (all from Sigma-Aldrich, Saint Louis, MO, USA). A549 cells were maintained in F-12K Medium (ATCC^®^, 30-2004) also supplemented with 10% FBS and 1% streptomycin/penicillin solution. HMEC-1 and HUVEC were cultured in Vascular Cell Basal Medium (ATCC^®^, PCS-100-030™) supplemented with Endothelial Cell Growth Kit-BBE (ATCC^®^, PCS-100-040™). All cells were maintained in a humidified incubator at 37 °C with 5% CO_2_ (HeraCell 150i, Thermo Fisher Scientific™, Waltham, MA, USA). Culture media were changed twice a week. Cells were passaged using a 0.025% trypsin-EDTA solution (Sigma-Aldrich) when confluency reached approximately 70%.

### 4.3. Immunohistochemistry (IHC)

Cancerous (LSCCs, *n* = 400; LUADs, *n* = 400) and non-malignant lung tissue samples (NMLT, *n* = 55) were fixed in 10% buffered formalin and embedded in paraffin for immunohistochemistry (IHC) assays. The following antibodies were used for the immunohistochemical examination of the markers: rabbit polyclonal antibody against SOX7 (1:500; ab94397, Abcam, Cambridge, UK), rabbit monoclonal antibody against SOX17 (1:400, ab224637, Abcam, Cambridge, UK), mouse monoclonal antibody against SOX18 (1:500, sc-166025, SantaCruz, Dallas, TX, USA), anti-SOX30 rabbit polyclonal antibody (1:200, NBP1-86503, NovusBio, Centennial, CO, USA), anti-MEF2C rabbit polyclonal antibody (1:200, HPA005533, SigmaAldrich, Louis, MO, USA), anti-VCAM1 rabbit polyclonal antibody (1:200, 11444-1-AP, ProteinTech, Rosemont, IL, USA) and anti-pSTAT3 mouse monoclonal antibody (1:200, #9145, Cell Signaling, Danvers, MA, USA). IHC was conducted using the Autostainer Link 48 (DakoCytomation, Glostrup, Denmark) to ensure consistent and reproducible results.

### 4.4. RNA Extraction, cDNA Synthesis, and Real-Time PCR Reactions

Total RNA extraction was performed using the RNeasy Mini Kit (Qiagen, Hilden, Germany) and subsequently transcribed into cDNA with the iScript cDNA Synthesis Kit (Bio-Rad Laboratories, Hercules, CA, USA) following the manufacturer’s protocol. The RT-qPCR was executed in 20 µL reactions on a 7500 Real-time PCR System (Applied Biosystems, Foster City, CA, USA), utilizing iTaq Universal Probes Supermix (Bio-Rad Laboratories, Hercules, CA, USA). The experiment employed TaqMan-specific probes from Applied Biosystems: Hs00846731_s1 for *SOX7*, Hs00751752_s1 for *SOX17*, Hs00746079_m1 for *SOX18*, Hs01555962_m1 for *SOX30*, Hs00231149_m1 for *MEF2C*, Hs01003372_m1 for *VCAM1*, Hs00374280_m1 for *STAT3*, and Hs00188166_m1 for *SDHA* as the reference gene. Each reaction was conducted in triplicate under the conditions: polymerase activation at 50 °C for 2 min, initial denaturation at 94 °C for 10 min, followed by 40 cycles of 94 °C for 15 s and 60 °C for 1 min. The relative mRNA expression levels were calculated using the Ct method.

### 4.5. SDS-PAGE and Western Blotting

A total of 100 paired non-small-cell lung cancer (NSCLC) tissue samples were analyzed, including 50 pairs of lung adenocarcinoma (LUAD) and matched normal lung tissue (NMLT), and 50 pairs of lung squamous cell carcinoma (LSCC) and matched LUAD tissue. All samples were lysed on ice using T-PER Tissue Protein Extraction Reagent (Thermo Fisher Scientific, Waltham, MA, USA), with an inhibitor cocktail (Sigma, St. Louis, MO, USA), 250 units of Benzonase^®^ (Merck Millipore, Bedford, MA, USA), and 2 mM PMSF. Similarly, cell lines were lysed on ice using Cell Lysis Buffer (Thermo Fisher Scientific, Waltham, MA, USA) with the same inhibitor cocktail, Benzonase^®^, and PMSF. Lysates containing 30 µg of total protein were mixed with 4× SDS-PAGE gel loading buffer (200 mM Tris–HCl, pH 6.8; 400 mM DTT; 8% SDS; 0.4% bromophenol blue; 40% glycerol), loaded onto 10% acrylamide gels, and separated by SDS-PAGE under reducing conditions. Proteins were then transferred to PVDF membranes. Post-transfer, the membranes were blocked for 1 h at room temperature in 4% BSA in TBST buffer, followed by overnight incubation at 4 °C with primary antibodies: anti-SOX7 (1:500), anti-SOX17 (1:400), anti-SOX18 (1:500), anti-SOX30 (1:500), anti-MEF2C (1:400), and anti-VCAM1 (1:400), and anti-pSTAT3 (1:400), The membranes were then washed with TBST buffer and incubated for 1 h at room temperature with HRP-conjugated anti-rabbit and anti-mouse secondary antibodies, diluted 1:3000 (Jackson ImmunoResearch, Mill Valley, CA, USA). After washing, the membranes were treated with the Immun-Star HRP Chemiluminescence Kit (Bio-Rad). Rabbit anti-human β-actin monoclonal antibody (#4970; Cell Signaling Technology, Danvers, MA, USA), diluted 1:1000, was used as the internal control. Western blotting results were analyzed using the ChemiDoc MP system (Bio-Rad) with Image Lab software version 6.0.1.

### 4.6. Spatial Transcriptomics for NSCLC Tissue Profiling

To investigate the spatial expression patterns of key regulatory genes—SOX7, SOX17, SOX18, SOX30, MEF2C, VCAM1, and p-STAT3—we performed spatial transcriptomic profiling in two major subtypes of non-small-cell lung cancer (NSCLC): lung adenocarcinoma (LUAD) and lung squamous cell carcinoma (LSCC). Our study focused on capturing the transcriptional heterogeneity across three defined tissue regions: the tumor core, tumor border, and adjacent non-malignant lung tissue.

Spatial transcriptomic analysis was conducted using the 10x Genomics Visium platform, adhering to the manufacturer’s protocols, and analyzed with Loop Browser software (10x Genomics, version 2.0).

Once the samples passed quality control, additional sections (5 μm) were cut using a Leica RM2255 microtome (Leica Microsystems, Wetzlar, Germany) and rehydrated in a 42 °C water bath before mounting onto Visium Spatial Gene Expression Slides (PN-2000233). Sections were dried in a desiccation chamber (Sanplatec, Osaka, Japan) at 42 °C for 3 h and then deparaffinized using Qiagen Deparaffinization Solution at 60 °C for 2 h. H&E staining was repeated in accordance with 10x Genomicstechnical specifications (CG000409), and high-resolution images were captured using both Sysmex Panoramic MIDI II (Sysmex Europe GmbH, Norderstedt, Germany).

Tissue sections were then processed using the CytAssist instrument from 10x Genomics (10x Genomics, Pleasanton, CA, USA), which enabled precise transfer of spatial information to capture slides. After decrosslinking (per protocol CG000407), sections were hybridized with the Visium Human Transcriptome Probe Set v1.0, targeting over 20,000 genes. Following hybridization, transcript capture, and RNase treatment, cDNA libraries were prepared using the Visium Library Preparation protocol and barcoded with the Dual Index Kit TS, Set A (PN-1000251).

### 4.7. Bisulfite Conversion of DNA and Droplet Digital Methylation-Specific PCR (ddMSP)

Genomic DNA was isolated from 5 μm thick FFPE tissue sections obtained from 15 LUAD and 15 LSCC cases. For each sample, laser capture microdissection (LCM; Leica, Germany) was used to selectively collect tissue from three distinct regions: the tumor core, the tumor border, and the adjacent non-malignant lung tissue (NMLT). DNA was isolated from all regions using the QIAamp DNA FFPE Tissue Kit (Qiagen), following the manufacturer’s protocol. To assess DNA methylation status, bisulfite conversion was performed using the EpiTect Plus DNA Bisulfite Kit (Qiagen). The concentration of bisulfite-treated DNA was quantified using the Qubit ssDNA Assay Kit (Thermo Fisher Scientific). CpG island sequences within the SOX18 and SOX30 gene regions were selected and retrieved from the UCSC Genome Browser (http://genome.ucsc.edu/, accessed on 21 October 2024) and analyzed using MethPrimer 2.0 (http://www.urogene.org/methprimer2, accessed on 14 March 2025) [36].

Custom primers and TaqMan probes for methylation-specific detection were designed and then synthesized by Merck Chemicals (Merck KGaA, Darmstadt, Germany). For each sample, about 10 to 40 ng of bisulfite-treated DNA was used in the droplet digital PCR (ddPCR) reaction. To measure unmethylated SOX18 and SOX30 CpG regions, we used a VIC-labeled TaqMan probe, while a FAM-labeled TaqMan probe was used to detect the methylated regions of these genes. The ddPCR was performed following the manufacturer’s guidelines.

Each PCR reaction was prepared in a final volume of 25 µL, which included ddPCR Supermix for Probes (no dUTP) from Bio-Rad, primers at 900 nM, and probes at 250 nM. Primer and probe sequences are provided in Table 3. Droplets were generated by mixing 20 µL of this reaction mix with 70 µL of droplet generation oil in a QX200 DG cartridge and processed using the QX200 Droplet Generator (both Bio-Rad instruments).

PCR amplification was carried out on a C100 Touch Thermal Cycler (Bio-Rad) with the following cycling conditions: enzyme activation for 10 min at 95 °C, followed by 40 cycles of 30 s at 94 °C (denaturation) and 1 min at 55 °C (annealing/extension). The reaction ended with a 10 min enzyme deactivation step at 98 °C. After PCR, plates were stored at 10 °C until droplet reading.

Droplets were then analyzed using the Bio-Rad QX200 Droplet Reader. Each sample was run in triplicate, and no-template controls were included in every run to check for contamination.

### 4.8. Adenovirus Transduction of Cell Lines

The adenoviral particles: AdmRa-hsa-miR-24 Viru and AmiRa Control particles used for transduction were bought from ABM (abm, Vancouver, BC, Canada). The cells were seeded in a 6 well plate at 70% co-fluency one day prior to transduction. After 24 h the cells were covered with 1 mL per well of viral culture supernatant for one hour in an incubator. The MOI used in the experiment for was 10. After one hour of incubation the media containing the virus were removed and replace with fresh complete media. The effectiveness of the transduction was determined on the mRNA and protein level using RT-qPCR, WB methods and confocal microscopy Olympus FV10i (Olympus Corporation, Hachioji, Tokyo, Japan).

### 4.9. Spheroids Formation

Three-dimensional (3D) spheroids were generated using human umbilical vein endothelial cells (HUVECs) in combination with non-small-cell lung cancer (NSCLC) cell line A549. Two cell lines were established and used: control cells and cells transduced with adenovirus expressing hsa-miR-24-3p. A549 and HUVEC were co-seeded with in a low adhesion 96-well plate (3D PrimeSurface^®^ 96 V, MS-9096VZ, Akita Sunitomo Bakelite, Akita, Japan) [37,38]. Before seeding, cells were counted, mixed 1:1 and centrifuged (300× *g* for 10 min) using Sigma 3–18K (Sigma-Aldrich^®^) [39]. Each well contained 5 × 10^3^ cells of each cell type in 100 µL of the appropriate cell-specific growth medium. Cultures were maintained for ten days with media changes performed twice a week. After the culture period, spheroids were collected for further analyses. For immunohistochemistry (IHC) spheroids were stained in haematoxylin for 4 min and fixed in cold 4% paraformaldehyde (PFA) and stored in 4 °C overnight. Subsequently, spheroids were dehydrated in a series of alcohol solutions with increasing concentrations and then embedded in paraffin blocks. Paraffin sections were prepared using standard histological protocols. In parallel, spheroids prepared for Western blot and RT-qPCR analyses were stored at −80 °C.

### 4.10. Statistical Analysis

The Shapiro–Wilk test was performed to assess the normality assumption of the groups investigated. The Wilcoxon signed-rank test was used to examine differences between the NSCLC and NMLT groups. In addition, the Spearman correlation test was used to analyze the existing relationships. Prism 8.1.0 (GraphPad Software, La Jolla, CA, USA) was used for all statistical analyses. Results were considered statistically significant at *p*  <  0.05.

## 5. Conclusions

This study provides a comprehensive characterization of SOX family transcription factors and their associated regulatory networks in non-small-cell lung cancer (NSCLC). Through integrative analysis combining immunohistochemistry, transcriptomics, methylation profiling, and functional assays, we demonstrate that SOX proteins play divergent roles in tumor biology, with implications for both disease progression and therapeutic targeting.

Our findings identify SOX7 and SOX30 as likely tumor suppressors, exhibiting reduced expression in NSCLC tissues, often through epigenetic silencing. In contrast, SOX17 and SOX18 emerge as context-dependent oncogenic drivers, associated with pro-tumorigenic pathways such as angiogenesis, inflammation, and immune modulation. Notably, SOX18 appears to function as a central node within a transcriptional network involving MEF2C, p-STAT3, and VCAM1—particularly in the tumor vasculature and invasive front—underscoring its relevance to tumor microenvironment remodeling.

We also uncover a novel regulatory axis involving miR-24-3p, which suppresses SOX18 expression and modulates other network components, suggesting potential for miRNA-based therapeutic intervention. The observed discrepancy between SOX18 promoter hypermethylation and elevated protein expression highlights the importance of considering multi-layered regulatory mechanisms in NSCLC pathogenesis.

Our study positions SOX transcription factors as both biomarkers and potential therapeutic targets in lung cancer, particularly within tumor–stroma interactions and vascular niches. These insights lay the groundwork for future in vivo studies aimed at therapeutic modulation of SOX-dependent pathways and underscore the value of integrating spatial, molecular, and functional data in cancer research.

## Figures and Tables

**Table 1:** Distribution of immunohistochemical expression of SOX7, SOX17, SOX18, SOX30, MEF2C, VCAM1, and p-STAT3 across NMLT, LSCC, and LUAD samples. Data are presented as the number of positive cases (*n*) and percentage (%). The *p*-values represent the results of Fisher’s exact test comparing NMLT vs. LSCC and NMLT vs. LUAD for each marker and subcellular localization.

		NMLT		LSCC		*p*	LUAD		*p*
		*n* = 55		*n* = 400		NMLT vs. LSCC	*n* = 400		NMLT vs. LUAD
		*n*	%	*n*	%		*n*	%	
**SOX7**	**Nuclear**	0	0.00	137	34.25	7.69 × 10^−10^	245	61.25	4.28 × 10^−21^
	**Cytoplasmic**	0	0.00	97	24.25	1.53 × 10^−6^	148	37.00	1.43 × 10^−10^
	**Endothelial cells**	11	20.00	256	64.00	7.81 × 10^−10^	240	60.00	2.63 × 10^−8^
**SOX17**	**Nuclear**	0	0.00	186	46.50	2.54 × 10^−14^	287	71.75	2.19 × 10^−27^
	**Cytoplasmic**	0	0.00	87	21.75	5.07 × 10^−6^	165	41.25	3.94 × 10^−12^
	**Endothelial cells**	17	43.50	174	43.50	8.18 × 10^−2^	233	58.25	1.50 × 10^−4^
**SOX18**	**Nuclear**	0	0.00	273	68.25	4.25 × 10^−25^	319	79.75	1.22 × 10^−33^
	**Cytoplasmic**	0	0.00	27	6.75	6.05 × 10^−2^	32	8.00	2.26 × 10^−2^
	**Endothelial cells**	37	67.27	230	57.50	1.90 × 10^−1^	334	83.50	8.34 × 10^−3^
**SOX30**	**Nuclear**	0	0.00	31	7.75	2.28 × 10^−2^	63	15.75	2.49 × 10^−4^
	**Cytoplasmic**	0	0.00	13	3.25	3.82 × 10^−1^	19	4.75	1.48 × 10^−1^
	**Endothelial cells**	0	0.00	0	0.00	1.00 × 10^0^	0	0.00	1.00 × 10^+0^
**MEF2C**	**Nuclear**	2	3.63	273	68.25	1.47 × 10^−21^	345	86.25	3.62 × 10^−36^
	**Cytoplasmic**	5	9.10	43	10.75	8.19 × 10^−1^	63	15.75	2.30 × 10^−1^
	**Endothelial cells**	25	45.45	323	80.75	8.90 × 10^−8^	364	91.00	2.42 × 10^−14^
**VCAM1**	**Nuclear**	1	1.81	12	3.00	1.00 × 10^0^	23	5.75	3.38 × 10^−1^
	**Cytoplasmic**	2	3.64	223	55.75	4.22 × 10^−15^	341	85.25	4.83 × 10^−35^
	**Endothelial cells**	29	52.72	313	78.25	1.04 × 10^−4^	349	87.25	1.42 × 10^−8^
**p-** **STAT3**	**Nuclear**	9	16.36	237	59.25	1.48 × 10^−9^	306	76.50	4.40 × 10^−18^
	**Cytoplasmic**	3	5.45	92	23.00	1.32 × 10^−3^	145	36.25	8.09 × 10^−7^
	**Endothelial cells**	18	32.72	87	21.75	8.68 × 10^−2^	123	30.75	7.58 × 10^−1^

**Table 2:** Clinicopathological characteristics of the non-small-cell lung cancer cases included in the study.

Characteristic	No. (%) of Patients (*n* = 800)
Age (years)	
Mean	66 ± 7.86
Range	43–88
Gender	
Male	442 (55.25)
Female	358 (44.75)
Histology	
Adenocarcinoma	400 (50.00)
Squamous	400 (50.00)
Tumor size	
T1 (<2 cm)	183 (22.88)
T2 (2–5 cm)	366 (45.75)
T3 (>5 cm)	167 (20.88)
T4	84 (10.50)
Lymph nodes	
N0	489 (61.13)
N1, N2, N3	311 (38.88)
pTNM	
1A	138 (17.25)
1B	165 (20.63)
2A	82 (10.25)
2B	79 (9.88)
3A	113 (14.13)
3B	125 (15.63)
4	98 (12.25)
Grade	
G1	174 (21.75)
G2	413 (51.63)
G3	213 (26.63)

**Table 3:** Sequences of the primer pairs and probes used in each assay for the methylation analysis of SOX18 and SOX30 CpG regions.

**SOX18 I**	FM primer	GCGGGGAACGGCAACCAGCG
	RM primer	CGGAATCCCGCCCGGCCTGA
	FU primer	GGTTTTGGTTTTTTGTTTTGGTT
	RU primer	AACAAATAAAACTAACAAATTCA
	Island M	FAM-GCTGTGCGCGGGGGAGGCCT
	Island U	VIC-CGGAATCCCGCCCGGCCTGA
**SOX18 II**	Left M primer	GCCCGGCCCGAGGCCACCGCC
	Right M primer	CCGCCCTCCCGGCCTGGCCT
	Left U primer	TTGGGTTGTTAGTTTTGGTGTT
	Right U primer	ACTACTTAAAAATAATCAAATAAA
	Island M	FAM-GCCCGGCCCGAGGCCACCGCC
	Island U	VIC-TCCCGGCCTGGCCTGCCCTT
**SOX18 III**	Left M primer	GCGGGGAGGTGGGGGGGCTG
	Right M primer	CGGAATCCCGCCCGGCCTGA
	Left U primer	GGTTTTGGTTTTTTGTTTTGGTT
	Right U primer	AACAAATAAAACTAACAAATTCA
	Island M	FAM-GCTGTGCGCGGGGGAGGCCT
	Island U	VIC-CGGAATCCCGCCCGGCCTGA
**SOX30 I**	Left M primer	CGTTACCCTGGTAACCGTGAC
	Right M primer	CGCTCACCGATGGAAATTCC
	Left U primer	TGTTATTTTGGTAATTTGTGAT
	Right U primer	AAATACAAATAAAATAAATAAACC
	Island M	FAM-TGGCTACCGCGTTACCCTGGTAA
	Island U	VIC-GTCCTTCCTGCAAGTCCTCC
**SOX30 II**	Left M primer	GGAAGCGCTGGCGCATCGC
	Right M primer	GAGTCGAGCTCGCTCACCG
	Left U primer	GGAAATTGTTGTTGTTTGT
	Right U primer	AAAATAAATAATAACCAAAAATAA
	Island M	FAM-ATTCCCCGGAAGCGCTGGCG
	Island U	VIC-CCGATGGAAATTCCGGCCGT
**SOX30 III**	Left M primer	AGCACAGACGTTAAGTATTGC
	Right M primer	CGAAGGCTTACACAGACTTCT
	Left U primer	AGTATAGTATGTTTAGTTTGT
	Right U primer	AAAATTACTTATTTTATAAAATAAA
	Island M	FAM-GTTAAGTATTGCCCACAAGCA
	Island U	VIC-TTCAAAGATATCTAACAGAATA

## Data Availability

The original contributions presented in this study are included in the article. Further inquiries can be directed at the corresponding author.
